# Association between Metabolic Score for Visceral Fat and the risk of hypertension in different ethnic groups: a prospective cohort study in Southwest China

**DOI:** 10.3389/fendo.2024.1302387

**Published:** 2024-03-18

**Authors:** Fuyan Zhang, Yiying Wang, Jie Zhou, Lisha Yu, Ziyun Wang, Tao Liu, Yangwen Yu

**Affiliations:** ^1^School of Public Health, the Key Laboratory of Environmental Pollution Monitoring and Disease Control, Ministry of Education, Guizhou Medical University, Guizhou, China; ^2^Guizhou Province Center for Disease Prevention and Control, Chronic Disease Prevention and Cure Research Institute, Guiyang, China

**Keywords:** METS-VF, visceral fat, hypertension, ethnicity, prospective cohort study

## Abstract

**Objective:**

Visceral adipose tissue assessment holds significant importance in hypertension prevention. This study aimed to explore the association between the Metabolic Score for Visceral Fat (METS-VF), a new indicator based on laboratory and anthropometry measures, and hypertension risk and to further investigate the association between the METS-VF and the risk of hypertension in different ethnic groups.

**Methods:**

In this study, a total of 9,280 people from 48 townships in 12 districts (counties) of Guizhou Province were selected for the survey using a multistage cluster random sampling method, and 5,127 cases were finally included in the analysis after excluding those with missing relevant data, losing visits, dying at follow-up, those who suffered from hypertension at baseline, and those whose information on the outcome of hypertension was not clear. Cox proportional hazard models were used to estimate hazard ratios (HRs) and 95% confidence intervals (95% CIs) between METS-VF and incident hypertension, and an accelerated failure time (AFT) model was applied to analyze the association between METS-VF and the onset time of hypertension.

**Results:**

The total person-years (PYs) of the 5,127 subjects were 36,188.52 years, and the median follow-up time was 6.64 years. During follow-up, 1,127 patients were newly diagnosed with hypertension, and the incidence density was 31.14/1,000 PYs. After adjusting for multivariables, compared with the METS-VF first (Q1), the third (Q3) and fourth (Q4) groups of the METS-VF increased by 29.9% and 61.5%, respectively (HR = 1.299 [1.061, 1.590] and 1.615 [1.280, 2.036]). The risk of hypertension increased with higher METS-VF values (HR = 1.323 [1.167, 1.500], *p*_trend_ < 0.001). In the Han Chinese population, Q2 and Q3 increased the risk of hypertension (HR = 1.459 [1.111, 1.917], 1.999 [1.417, 2.718]), and the onset of hypertension was advanced by 0.653 (β = −0.653 (−0.930, −0.375]) years for per 1 unit increase in METS-VF. However, these associations were not found in ethnic minorities.

**Conclusion:**

METS-VF was significantly positively associated with the risk of hypertension, and the association was different among ethnic groups.

## 1 Introduction

Hypertension is one of the most important risk factors for cardiovascular and cerebrovascular diseases, chronic kidney disease, and dementia. Elevated blood pressure is a major preventable risk factor for cardiovascular death and the global burden of disease in most regions of the world (1). It is estimated that hypertension will affect more than 1.5 billion people worldwide by 2025, and the estimated number of deaths related to blood pressure has increased to 49%, to 10.4 million each year (2). Hypertension has become a major public health problem facing the world, and curbing hypertension effectively has become a consensus (3, 4). In recent years, studies have found that the accumulation of visceral adipose tissue (VAT) is associated with insulin resistance (IR) and the risk of hypertension (5–8). The Metabolic Score for Visceral Fat (METS-VF) (9), which combines the Metabolic Index of Insulin Resistance (METS-IR), waist-to-height ratio, age, and gender to estimate visceral and subcutaneous fat, is mostly used to predict chronic diseases such as hypertension and diabetes mellitus (10–13). One study found that high METS-VF is significantly associated with increased risk of hypertension in rural Chinese populations but did not examine it in urban populations (13). Published studies assessing the association between METS-VF and the risk of hypertension based on cohort studies are rare. In particular, it has not been reported on the differences between different ethnic groups. Guizhou, located in southwest China, is a multiethnic province, with more than 36.44% of the whole province’s population being ethnic minorities, including the Miao, Buyi, Dong, and so forth (14). Therefore, this study aims to explore the association between METS-VF and the ethnic differences in hypertension based on a prospective cohort study.

## 2 Methods

### 2.1 Study population

The data were obtained from the Guizhou Population Health Cohort Study (GPHCS), a large population database that aimed to investigate the prevalence of chronic diseases and risk factors. In 2010, a baseline survey was conducted on 9,280 adult permanent residents in 12 districts (counties) of Guizhou Province using a multistage cluster random sampling method. From 2016 to 2020, 8,163 individuals completed the follow-up survey. During the follow-up, information was updated on the status of major chronic diseases and vital status, with a response rate of 88.00% (15). This study was approved by the Institutional Review Board of Guizhou Province Centre for Disease Control and Prevention (No. s2017-02), and written informed consent was signed by all subjects.

**Figure 1:**
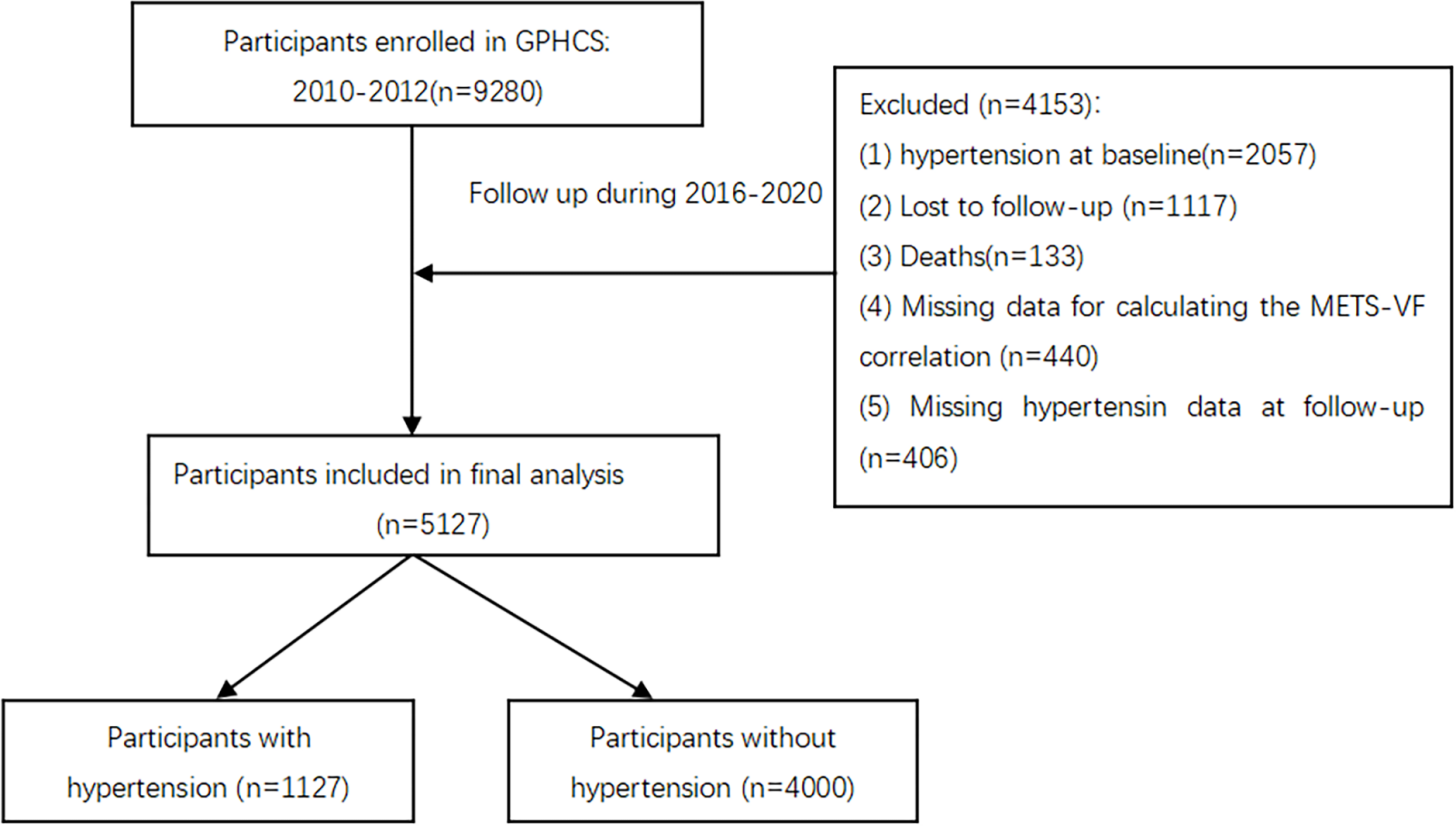
The flow chart of the study.

To explore the relationship between METS-VF and hypertension, we excluded participants with missing data (*n* = 440), loss to follow-up (*n* = 1,117), follow-up deaths (*n* = 133), baseline hypertension (*n* = 2,057), and unclear information on hypertension outcomes (*n* = 406). Finally, a total of 5,127 participants were included in this study (**Figure 1**). All deaths were confirmed through the death registration information system and the basic public health service system.

### 2.2 Data collection

Baseline information included sociodemographic characteristics (age, gender, ethnicity, education attainment, residence, marital status, and occupation), lifestyle (tobacco and alcohol consumption, physical activity), and medical history of chronic noncommunicable diseases (hypertension, dyslipidemia, diabetes mellitus, and cardiovascular diseases), which was collected by trained investigators through structured questionnaires via face-to-face interview.

Physical examination data, including height, weight, waist circumference, and blood pressure, were collected by trained investigators through standard procedures. Standing height was measured without shoes using a unified height meter (accuracy is 0.1 cm). Weight was measured using an electronic weight scale (accuracy is 0.1 kg). WC was measured using a waist ruler (accuracy is 0.1 cm) at the midpoint between the lowest rib cage and the iliac crest. Blood pressure data were taken as the average of three consecutive measurements. Venous blood samples were obtained in the early morning for fasting blood glucose, total cholesterol, high-density lipoprotein cholesterol, low-density lipoprotein cholesterol, and triglyceride levels after the participants had fasted for at least 8 h.

The above methods for data collection were identical in the baseline and follow-up studies.

### 2.3 Assessment of hypertension and METS-VF

Participants were diagnosed with hypertension if they met either of the following two criteria: self-reported diagnosis of hypertension or antihypertensive treatment by physicians; or systolic blood pressure (SBP) ≥ 140 mmHg and/or diastolic blood pressure (DBP) ≥ 90 mmHg (16). The blood pressure was measured with the same type of electronic sphygmomanometer (accuracy is 0.1 mmHg). Before measuring blood pressure, one should take a proper rest for 3–5 minutes and avoid strenuous exercise, eating, smoking, and drinking stimulant beverages, such as coffee, strong tea, wine, etc. If the difference in the three measurements did not exceed 10 mmHg, the average of the three measurements was taken as the final reading; if the difference between the three measurements was large, the average of the two similar measurements was taken as the final reading; if only one measurement was taken, the final reading was taken directly.

$$\begin{array}{c} \text{METS}-\text{VF}=4.466\;+\;0.011*[{\left(\text{Ln}(\text{METS}-\text{IR})\right)}^{3}]\\ \;\;\;\;\;+3.239*[{\left(\text{Ln}(\text{WHTR})\right)}^{3}\;+0.319(\text{sex})\\ \;\;\;\;\;+0.594*(\text{Ln}(\text{age}))]\{\text{male}=1,\;\text{female}=0\} \end{array}\;$$

(9)

$$\begin{array}{c} \text{METS}-\text{IR}=(\text{Ln}((2*\text{FPG}(\text{mg}/\text{dL}))\\ \;\;\;\;+\text{TG}(\text{mg}/\text{dL}))*\text{BMI}(\text{kg}/{\text{m}}^{2})/(\text{Ln}(\text{HDL}\\ \;\;\;\;-\text{C}(\text{mg}/\text{dL})) \end{array}\;$$

(17)

### 2.4 Assessment of covariates

Diabetes was defined as meeting one of the following conditions: having been diagnosed with diabetes by township or community and above hospitals, fasting plasma glucose (FPG) ≥ 7.0 mmol/L (126 mg/dL), 2-h postmeal blood glucose ≥ 11.1 mmol/L (200 mg/dL) (18). Dyslipidemia was diagnosed as meeting one of the following conditions: total cholesterol (TC) ≥ 6.22 mmol/L; triglycerides (TG) ≥ 2.26 mmol/L; high-density lipoprotein cholesterol (HDL-C) < 1.04 mmol/L; low-density lipoprotein cholesterol (LDL-C) ≥ 4.14 mmol/L; self-reported physician dyslipidemia diagnosis or having received lipid-lowering treatment (19). Cardiovascular disease (CVD) was defined as meeting one of the following conditions: self-reported diagnosis by a physician; death based on myocardial infarction/cerebral hemorrhage/cerebral infarction, etc. Body mass index (BMI) was calculated as weight in kg divided by height in m squared, low weight: BMI kg/m^2^ < 18.5, normal weight: 18.5 kg/m^2^ ≤ BMI < 24 kg/m^2^, overweight: 24 kg/m^2^ ≤ BMI < 28 kg/m^2^, and obesity: BMI ≥ 28 kg/m^2^ (20). Smoking was defined as still smoking at the time of the survey, including daily smoking and occasional smoking at the time of the survey.

### 2.5 Statistical analysis

The Statistical Package for the Social Sciences (Version 26.0; IBM Corporation, Armonk, NY, USA) and R software (Version 4.2.3; R Foundation for Statistical Computing, Vienna, Austria) were used to perform statistical analyses. The continuous variables that do not obey the normal distribution are expressed as medians (second quartile, third quartile), and categorical variables are described by frequencies and percentages. Baseline characteristics were compared using the Wilcoxon rank sum test or the Chi-square test. Person-years were used as the time variable. The person-years were calculated from the baseline survey to the onset of hypertension or the end of follow-up, and the incidence density of different METS-VF groups was calculated.

The METS-VF values were divided into four groups by quartiles, ≤ 5.15 (Q1), 5.16~5.64 (Q2), 5.65~6.06 (Q3), and ≥ 6.07 (Q4), with Q1 as the reference level. We fitted three Cox proportional hazard regression models to estimate the hazard ratio (HR), the adjusted HR (aHR), and corresponding 95% confidence interval to determine the association between METS-VF and the risk of hypertension and performed the subgroup analysis according to the ethnicity. Model 1: without any adjustment for covariates. Model 2: adjusted for age (18–44 years, 45–60 years, ≥ 60 years), gender (man, woman), ethnicity (Han Chinese, minority), region (urban, rural). Model 3: model 2 added education level (below primary, primary, junior, high/technical, college/above), marital status (single, married/cohabitation, divorce/widowed/separation), smoking (current smoking, nonsmoking), alcohol consumption (yes, no), sleep duration (< 7/> 9 h, 7–9h), sedentary time (< 6 h, ≥ 6 h), having diabetes mellitus (yes, no), dyslipidemia (yes, no), family history of hypertension (yes, no), BMI grade (light weight, normal, overweight, obesity), salt intake (< 25 g/day, ≥ 25 g/day), oil intake (< 5 g/day, ≥ 5 g/day). A restrictive cubic-like spline plot was used to plot the dose–response relationship of the baseline METS-VF values with the risk of hypertension. The AFT model was applied to evaluate the effect of METS-VF on the onset time of hypertension, and the logistic distribution was selected for the AFT model according to the minimum AIC information criterion in different survival distributions (i.e., Weibull, index, logistic, Gauss).

Sensitivity analysis was conducted by excluding participants who were diagnosed with baseline diabetes, dyslipidemia, cardiovascular disease (including coronary heart disease and stroke), and individuals with a single blood pressure measurement. All tests were conducted two-sided, and a *p*-value less than or equal to 0.05 was considered statistically significant.

## 3 Results

### 3.1 Baseline characteristics of participants

The total person-years of follow-up was 36,188.52 years. At baseline, 5,127 participants were included in the analysis, of which 2,353 (45.89%) were men, 2,774 (54.11%) were women, 2,974 (58.01%) were Han Chinese, and 2,153 (41.99%) were minority. During a median follow-up of 6.64 years, the incident density of 1,127 newly diagnosed hypertension was 31.14/1,000 PYs. The cumulative incidence of hypertension was 22.90% in Han Chinese and 20.72% in minority nationalities. In the Han Chinese population, hypertensive patients as compared with nonhypertensive patients, different regions, gender, age group, education level, marital status, family history of hypertension, history of diabetes, BMI grade, current smoking, oil intake, static time, and METS-VF, which were statistically significant (*p* < 0.05). There were statistically significant (*p* < 0.05) in terms of age, marital status, history of dyslipidemia, oil intake, and METS-VF between ethnic minorities with hypertension and those without (**Table 1**).

**Table 1:** Baseline characteristics for participants.

Characteristics	Subgroups	Participants (*n* = 5,127)	Total		*p*-value	Han Chinese		*p*-value	Minority		*p*-value
			Hypertension (*n* = 1,127)	Nonhypertension (*n* = 4,000)		Hypertension (*n* = 681)	Nonhypertension (*n* = 2,293)		Hypertension (*n* = 446)	Nonhypertension (*n* = 1,707)	
Region	Urban	1,814 (35.38)	365 (20.12)	1,449 (79.88)	0.017	1,202 (79.97)	301 (20.03)	< 0.001	247 (79.42)	64 (20.58)	0.949
	Rural	3,313 (64.62)	762 (23.00)	2,551 (77.00)		1,091 (74.17)	380 (25.83)		1,460 (79.26)	382 (20.74)	
Sex	Male	2,353 (45.89)	550 (23.37)	1,803 (76.63)	0.027	1,033 (75.07)	343 (24.93)	0.015	770 (78.81)	207 (21.19)	0.622
	Female	2,774 (54.11)	577 (20.80)	2,197 (79.20)		1,260 (78.85)	338 (21.15)		937 (79.68)	239 (20.32)	
Ethnic	Minority	2,153 (41.99)	446 (20.72)	1,707 (79.28)	0.062	–	–	–	–	–	–
	Ethnic Han	2,974 (58.01)	681 (22.90)	2,293 (77.10)		–	–		–	–	
Age	18 years~	3,152 (61.48)	517 (16.40)	2,635 (83.60)	< 0.001	1,550 (83.92)	297 (16.08)	< 0.001	1,085 (83.14)	220 (16.86)	< 0.001
	45 years~	1,407 (27.44)	396 (28.14)	1,011 (71.86)		552 (69)	248 (31)		459 (75.62)	148 (24.38)	
	60 years~	568 (11.08)	214 (37.68)	354 (62.32)		191 (58.41)	136 (41.59)		163 (67.63)	78 (32.37)	
Educational level	Below primary	1,697 (33.10)	478 (28.17)	1,219 (71.83)	< 0.001	567 (66.16)	290 (33.84)	< 0.001	652 (77.62)	188 (22.38)	0.077
	Primary	1,027 (20.03)	231 (22.49)	796 (77.51)		455 (77.38)	133 (22.62)		341 (77.68)	98 (22.32)	
	Junior	1,621 (31.62)	295 (18.20)	1,326 (81.8)		830 (81.69)	186 (18.31)		496 (81.98)	109 (18.02)	
	High/technical	518 (10.10)	82 (15.83)	436 (84.17)		283 (84.23)	53 (15.77)		153 (84.07)	29 (15.93)	
	College/above	264 (5.15)	41 (15.53)	223 (84.47)		158 (89.27)	19 (10.73)		65 (74.71)	22 (25.29)	
Marital status	Single	588 (11.47)	74 (12.59)	514 (87.41)	< 0.001	311 (86.87)	47 (13.13)	< 0.001	203 (88.26)	27 (11.74)	< 0.001
	Married/cohabitation	4,102 (80.01)	928 (22.62)	3,174 (77.38)		1,849 (76.40)	571 (23.60)		1,325 (78.78)	357 (21.22)	
	Divorce/widowed/separation	419 (8.17)	117 (27.92)	302 (72.08)		127 (67.91)	60 (32.09)		175 (75.43)	57 (24.57)	
	Other	18 (0.35)	8 (44.44)	10 (55.56)		6 (66.67)	3 (33.33)		4 (44.44)	5 (55.56)	
Family history of hypertension	Yes	520 (10.14)	98 (18.85)	422 (81.15)	0.053	311 (83.38)	62 (16.62)	0.005	111 (75.51)	36 (24.49)	0.183
	No	3,257 (63.53)	707 (21.71)	2,550 (78.29)		1,463 (76.76)	443 (23.24)		1,087 (80.46)	264 (19.54)	
	Unclear	1,350 (26.33)	322 (23.85)	1,028 (76.15)		519 (74.68)	176 (25.32)		509 (77.71)	146 (22.29)	
History of diabetes	No	4,813 (93.88)	1,049 (21.80)	3,764 (78.20)	0.207	2,180 (77.58)	630 (22.42)	0.010	1,584 (79.08)	419 (20.92)	0.395
	Yes	314 (6.12)	78 (24.84)	236 (75.16)		113 (68.90)	51 (31.10)		123 (82.00)	27 (18.00)	
History of dyslipidemia	No	2,437 (47.53)	517 (21.21)	1,920 (78.79)	0.207	1,027 (76.64)	313 (23.36)	0.589	893 (81.40)	204 (18.60)	0.013
	Yes	2,690 (52.47)	610 (22.68)	2,080 (77.32)		1,266 (77.48)	368 (22.52)		814 (77.08)	242 (22.92)	
BMI^a^	Lightweight	322 (6.29)	62 (19.25)	260 (80.75)	0.001	152 (81.72)	34 (18.28)	0.003	108 (79.41)	28 (20.59)	0.210
	Normal	3,381 (66.02)	707 (20.91)	2,674 (79.09)		1,468 (78.13)	411 (21.87)		1,206 (80.29)	296 (19.71)	
	Overweight	1,177 (22.98)	284 (24.13)	893 (75.87)		573 (75.30)	188 (24.70)		320 (76.92)	96 (23.08)	
	Obesity	241 (4.71)	74 (30.71)	167 (69.29)		96 (66.67)	48 (33.33)		71 (73.20)	26 (26.80)	
Smoking	No	3,751 (73.16)	785 (20.93)	2,966 (79.07)	0.003	1,650 (78.53)	451 (21.47)	0.004	1,316 (79.76)	334 (20.24)	0.327
	Yes	1,376 (26.84)	342 (24.85)	1,034 (75.15)		643 (73.65)	230 (26.35)		391 (77.73)	112 (22.27)	
Never drink	No	1,579 (30.80)	357 (22.61)	1,222 (77.39)	0.469	651 (76.05)	205 (23.95)	0.386	571 (78.98)	152 (21.02)	0.802
	Yes	3,548 (69.20)	770 (21.70)	2,778 (78.30)		1,642 (77.53)	476 (22.47)		1,136 (79.44)	294 (20.56)	
Oil intake^a^ (g/day)	< 25	1,474 (28.99)	284 (19.27)	1,190 (80.73)	0.003	668 (79.52)	172 (20.48)	0.044	522 (82.33)	112 (17.67)	0.024
	≥ 25	3,610 (71.01)	835 (23.13)	2,775 (76.87)		1,602 (76.07)	504 (23.93)		1,173 (77.99)	331 (22.01)	
Salt intake (g/day)	< 5	915 (17.85)	185 (20.22)	730 (79.78)	0.155	473 (80.44)	115 (19.56)	0.031	257 (78.59)	70 (21.41)	0.738
	≥ 5	4,212 (82.15)	942 (22.36)	3,270 (77.64)		1,820 (76.28)	566 (23.72)		1,450 (79.41)	376 (20.59)	
Sleep time (h/day)	< 7/> 9	1,087 (21.20)	255 (23.46)	832 (76.54)	0.185	496 (74.92)	166 (25.08)	0.131	336 (79.06)	89 (20.94)	0.898
	7–9	4,040 (78.80)	872 (21.58)	3,168 (78.42)		1,797 (77.72)	515 (22.28)		1,371 (79.34)	357 (20.66)	
Sedentary time (h/day)	< 6	586 (11.43)	105 (17.92)	481 (82.08)	0.012	332 (82.79)	69 (17.21)	0.004	149 (80.54)	36 (19.46)	0.659
	≥ 6	4,541 (88.57)	1,022 (22.51)	3,519 (77.49)		1,961 (76.21)	612 (23.79)		1,558 (79.17)	410 (20.83)	
METS-VF	–	5.97 (5.46, 6.43)	5.91 (5.41, 6.38)	6.17 (5.67, 6.61)	< 0.001	5.91 (5.38, 6.38)	6.26 (5.77, 6.68)	< 0.001	5.92 (5.44, 6.36)	6.05 (5.57,6.51)	< 0.001

a With missing value.

### 3.2 Associations of METS-VF with risk of hypertension

Without adjusting for any confounding variables, compared with Q1, Q2 (HR = 1.282 [1.062, 1.549]), Q3 (HR = 1.693 [1.414, 2.027]), and Q4 (HR = 2.432 (2.049, 2.886]) were associated with an increased risk of hypertension. In the fully adjusted model, the risk of hypertension in Q3 and Q4 populations was 1.299 times (HR = 1.299 (1.061, 1.590]) and 1.615 times (HR = 1.615 [1.280, 2.036]) higher than that of the Q1 population, respectively. The risk of hypertension also increased with higher METS-VF values (*p*_trend_ < 0.001). Interaction analysis showed that there was an interaction between ethnicity and METS-VF, *p* = 0.003. Further analysis of ethnic stratification in Model 3 showed that both Q2 and Q3 increased the risk of hypertension in the Han Chinese population (HR = 1.459 [1.111~1.917], 1.999 [1.417~2.718]). However, the association between METS-VF and the risk of hypertension was not statistically significant in the ethnic minority population (*p* > 0.05) (**Table 2**).

**Table 2:** COX regression analysis of METS-VF levels and different groups and the risk of hypertension.

	Cases (*n*)	Incidence density (1,000 PYs)	Model 1	*p*-value	Model 2	*p*-value	Model 3	*p*-value
			HR (95% CI)		HR (95% CI)		HR (95% CI)	
METS-VF level	1,127	31.14	1.638 (1.504, 1.784)	< 0.001	1.361 (1.243, 1.490)	< 0.001	1.323 (1.167, 1.500)	< 0.001
METS-VF group								
Q1	198	21.44	1.000		1.000		1.000	
Q2	238	25.98	1.282 (1.062, 1.549)	0.010	1.171 (0.969, 1.417)	0.103	1.108 (0.907, 1.353)	0.316
Q3	297	33.02	1.693 (1.414, 2.027)	< 0.001	1.390 (1.155, 1.672)	< 0.001	1.299 (1.061, 1.590)	0.011
Q4	394	44.78	2.432 (2.049, 2.886)	< 0.001	1.741 (1.451, 2.090)	< 0.001	1.615 (1.280, 2.036)	< 0.001
*p*_trend_				< 0.001		< 0.001		< 0.001
Han Chinese	681	32.11	1.831 (1.642, 2.042)	< 0.001	1.449 (1.288, 1.631)	< 0.001	1.493 (1.265, 1.761)	< 0.001
METS-VF								
Q1	106	19.25	1.000		1.000		1.000	
Q2	132	25.58	1.444 (1.118, 1.866)	0.005	1.291 (0.998, 1.671)	0.052	1.183 (0.901, 1.554)	0.226
Q3	178	34.51	2.041 (1.604, 2.597)	< 0.001	1.592 (1.242, 2.041)	< 0.001	1.459 (1.111, 1.917)	0.007
Q4	265	49.28	3.053 (2.436, 3.828)	< 0.001	1.999 (1.566, 2.552)	< 0.001	1.999 (1.471, 2.718)	< 0.001
*p*_trend_				< 0.001		< 0.001		< 0.001
Minority	446	29.76	1.318 (1.144, 1.518)	< 0.001	1.172 (1.013, 1.356)	0.033	1.038 (0.851, 1.265)	0.713
METS-VF								
Q1	92	24.67	1.000		1.000		1.000	
Q2	106	26.49	1.037 (0.785, 1.372)	0.796	0.986 (0.744, 1.306)	0.921	0.965 (0.717, 1.297)	0.812
Q3	119	31.01	1.250 (0.952, 1.641)	0.108	1.105 (0.837, 1.458)	0.481	1.046 (0.771, 1.418)	0.774
Q4	129	37.72	1.623 (1.242, 2.121)	< 0.001	1.323 (1.001, 1.748)	0.049	1.073 (0.747, 1.541)	0.703
*p*_trend_				< 0.001		< 0.001		0.656

Model 1: unadjusted; model 2: adjusted age, sex, ethnicity, and region; model 3: model 2 added education level, marital status, smoking, drinking, sleep duration, sedentary time, diabetes mellitus, dyslipidemia, family history of hypertension, BMI grade, salt intake, and oil intake.

### 3.3 Dose–response relationship between METS-VF and risk of incident hypertension

**Figure 2:**
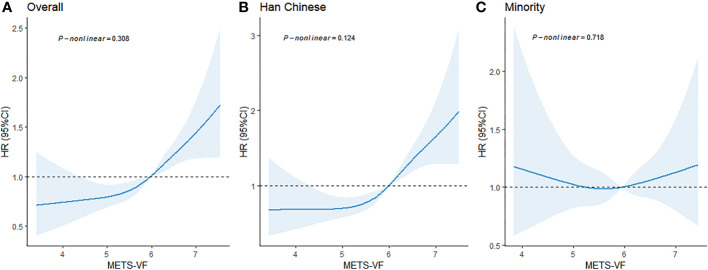
Dose–response relationship between baseline METS-VF levels and the risk of hypertension. **(A)** For the total population; **(B)** For the Han Chinese; **(C)** For the Minority. Adjusted age, sex, ethnicity, region, education level, marital status, smoking, alcohol consumption, sleep duration, sedentary time, diabetes mellitus, dyslipidemia, family history of hypertension, BMI grade, salt intake, and oil intake.

We employed a four-knot RCS regression model to fit the dose–response curves for METS-VF in relation to the risk of hypertension. The RCS analysis revealed that the association between METS-VF and hypertension risk was linear (*p* for nonlinearity = 0.308). Furthermore, as baseline METS-VF values increased, so did the risk of hypertension. However, this association has not been found among ethnic minorities (**Figure 2**).

### 3.4 Subgroup analysis

**Figure 3:**
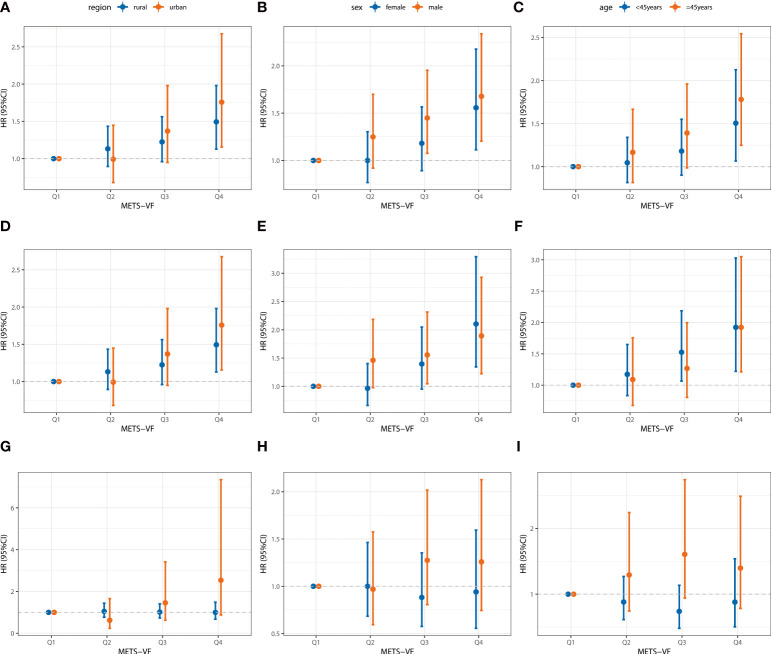
Association between baseline METS-VF and the incident risk of hypertension by region, sex, and age in different groups. **(A–C)** For the total population; **(D–F)** For the Han Chinese; **(G–I)** For the Minority. Adjusted age, sex, ethnicity, region, education level, marital status, smoking, alcohol consumption, sleep duration, sedentary time, diabetes mellitus, dyslipidemia, family history of hypertension, BMI grade, salt intake, and oil intake.

The participants were categorized into three subgroups according to gender, age group, and region. Compared with Q1, Q4 was associated with an increased risk of hypertension in different subgroups in the general population and the Han Chinese population (*p* < 0.05). However, the association between METS-VF subgroups and hypertension in ethnic minority populations was not statistically significant in any of the subgroups (*p* > 0.05) (**Figure 3**).

### 3.5 Associations of METS-VF with time to onset of hypertension

A significant positive association was observed between METS-VF at baseline and time to onset of hypertension during the follow-up survey (β = −0.425 [−0.630, −0.220]), that is, the onset of hypertension was advanced by 0.425 years per 1 unit increase in METS-VF. In the Han Chinese population, the onset time of hypertension reached 0.653 years earlier (β = −0.653 [−0.930, −0.375]). In ethnic minorities, we failed to detect a significant association (*p* > 0.05) (**Table 3**).

**Table 3:** Association between METS-VF and the onset of hypertension in the total population and different ethnic groups.

variable	Overall		Han Chinese		Minority	
	β (95%CI)	*p*-value	β (95%CI)	*p*-value	β (95%CI)	*p*-value
METS-VF level	−0.425 (−0.630, −0.220)	< 0.001	−0.653 (−0.930, −0.375)	< 0.001	−0.015 (−0.315, 0.285)	0.923
METS-VF group						
Q1	1		1		1	
Q2	−0.107 (−0.423, 0.208)	0.505	−0.267 (−0.705, 0.170)	0.232	0.121 (−0.317, 0.560)	0.586
Q3	−0.350 (−0.671, −0.029)	0.033	−0.601 (−1.043, −0.159)	0.007	0.025 (−0.428, 0.477)	0.915
Q4	−0.693 (−1.071, −0.315)	< 0.001	−1.090 (−1.603, −0.577)	< 0.001	−0.040 (−0.589, 0.508)	0.885

Adjusted age, sex, ethnicity, region, education level, marital status, smoking, alcohol consumption, sleep duration, sedentary time, diabetes mellitus, dyslipidemia, family history of hypertension, BMI grade, salt intake, and oil intake.

### 3.6 Sensitivity analysis

**Figure 4:**
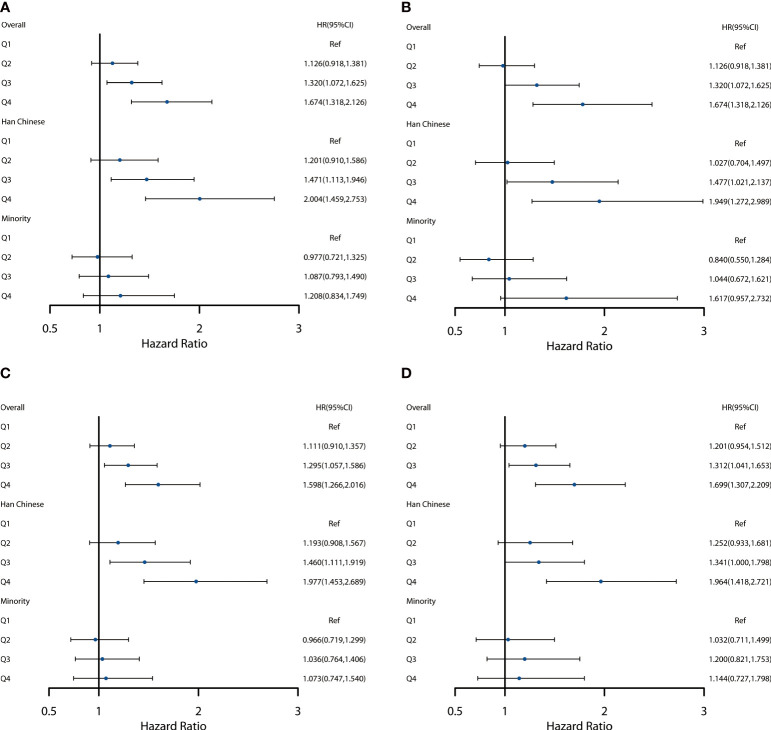
Sensitivity analysis of different METS-VF groups and risk of hypertension. **(A)** Excluding patients with diabetes mellitus at baseline. **(B)** Excluding patients with dyslipidemia at baseline. **(C)** Excluding patients with cardiovascular disease at baseline (including coronary heart disease and stroke). **(D)** Excluding participants with a single blood pressure measurement.

Four sensitivity analyses were conducted by excluding participants who were diagnosed with diabetes, dyslipidemia, and cardiovascular disease (including coronary heart disease and stroke) at baseline and individuals with a single blood pressure measurement. The results of which did not differ substantially from those of the primary analyses (**Figure 4**).

## 4 Discussion

Based on a prospective cohort study in Southwest China, METS-VF levels had a significant positive association with increased risk for hypertension. With every unit increase in METS-VF, the risk of hypertension increases by 32.3%. In addition, our study showed that the onset of hypertension was advanced with increasing METS-VF scores. Nevertheless, this association was not found in ethnic minorities. These findings indicated that it is crucial to pay attention to METS-VF for the prevention and control of hypertension, which provides a reference basis for future ethnic genetic research.

Currently, there is relatively limited research on the association between METS-VF levels and the risk of hypertension. Moreover, studies related to METS-VF and the risk of hypertension in different ethnic groups have not been reported. As far as we know, this was the first prospective cohort study aimed at exploring the risk of hypertension in different ethnic populations with METS-VF. Previous studies have demonstrated a positive association between METS-VF and hypertension. Feng et al. conducted a cohort study of 10,297 participants in rural China, which showed that the positive association between METS-VF and the risk of hypertension in Q2, Q3, and Q4 persisted (ORs [95% CI]: 1.66 [1.39–1.99], 2.50 [2.10–2.97], and 3.84 [3.23–4.56]) and progressively higher risk of hypertension with increasing quartiles of METS-VF (13). In another Mexican study, METS-VF was shown to predict cardiometabolic risk independently of BMI. Bello-Chavolla et al. reported that METS-VF levels were strongly associated with the risk of hypertension, with individuals in the highest quintile having a 3.7 times greater risk of hypertension than those in the lowest quintile (9). These are generally consistent with the findings of our study. There are several underlying association mechanisms between METS-VF and the risk of developing hypertension. Firstly, it may be that excess visceral fat deposition allows for a higher release of circulating free fatty acids, which can lead to atherosclerosis, hyperlipidemia, hypertension, and cardiovascular disease (21). Secondly, there is evidence that the accumulation of visceral adipose tissue is associated with impaired vascular health, adipocyte dysfunction, inflammation, and adipokine dysregulation. This promotes multiple vascular injuries, proinflammatory states, and glycolipid metabolism impairments, leading to a stress-induced response to IR (22). Thirdly, plasminogen activator inhibitor-1 may play a key role in visceral fat associated with the risk of hypertension. In the Framingham Offspring Study, plasminogen activator inhibitor-1 levels are associated with increased levels of systolic and diastolic blood pressure, which are the main circulating inhibitors of thrombolysis. More importantly, visceral fat produces more of this peptide than subcutaneous fat (23, 24).

Stratified analysis showed that METS-VF was able to significantly increase the risk of hypertension in the Han Chinese participants, but the association was not observed in ethnic minority participants. The possible reason is that given the differences in visceral fat levels among different races/ethnicities (25–27), it may lead to variations in the control of blood pressure by the renin–angiotensin–aldosterone system (28, 29). Some studies have shown that although visceral fat varies among different racial/ethnic groups, the effect of visceral fat on systolic and diastolic blood pressure is significant (30), which can increase the risk of hypertension (31). However, in this study, we did not find any association between METS-VF and the risk of hypertension in ethnic minority populations. There are two possible reasons to explain this. One reason may be that different ethnic populations have unique genetic characteristics for the same gene. The migration of ethnic groups is hindered by geographical barriers and influenced by culture, so there are different degrees of different food habits, cultural life, and genetic exchange between different peoples (32). Existing studies have shown that there are some genetic differences between Han and ethnic groups in Guizhou. For example, the allele and genotype frequencies of different single-nucleotide polymorphisms (SNP) of amyloid precursor protein (APP) are significantly different between Han and ethnic minorities in Guizhou (33). Another study investigated the relationship between single-nucleotide polymorphisms of the apolipoprotein E gene, rs725960, rs440446, rs769449, rs429358, rs7412, rs1065853, and rs439401, and essential hypertension (EH) in the Guizhou population and found that rs439401 was associated with the susceptibility to EH in the Guizhou Han population and may have ethnic specificity (34). Another is that differences in social support and lifestyle factors may also account for differences in the increased risk of hypertension between races/ethnicities (35, 36). In addition, relevant studies have shown that the characteristic diet of ethnic minorities can reduce blood lipid levels and improve lipid metabolism disorders and the oxidative stress caused by obesity (37–39). Even so, due to the lack of research on visceral fat and hypertension in ethnic minorities in China, the relationship between the METS-VF and the risk of incident hypertension needs to be further investigated to examine the mechanisms involved in ethnic differences.

Compared with previous studies, the strength of this research lies in its prospective design with clear characteristics and a longer follow-up period (up to 10 years). Our study was the first to investigate the association between METS-VF and hypertension risk among different ethnic groups in southwest China. However, there are also some limitations. First, despite the adjustment for multiple potential confounders, there may be other factors, such as regional environmental and socioeconomic characteristics, genetics, diet cultures, local customs, and psychological factors, affecting results. Second, we only conducted the baseline METS-VF study with the risk of incident hypertension. Finally, this study only investigated the permanent residents of Southwest China, and the extrapolation of the results was limited.

## 5 Conclusion

In conclusion, high METS-VF is significantly associated with an increased risk of hypertension, but this association was not found in ethnic minority populations. This result provides new evidence for the relationship between METS-VF and the risk of hypertension in southwest China and highlights the need to focus on visceral fat and hypertension prevention.

## Data availability statement

The raw data supporting the conclusions of this article will be made available by the authors, without undue reservation.

## Ethics statement

The studies involving humans were approved by Institutional Review Board of Guizhou Province Centre for Disease Control and Prevention. The studies were conducted in accordance with the local legislation and institutional requirements. The participants provided their written informed consent to participate in this study.

## Author contributions

FZ: Writing – original draft. YW: Writing – original draft. JZ: Writing – review & editing. LY: Writing – review & editing. ZW: Writing – original draft. TL: Writing – review & editing. YY: Writing – review & editing.
